# Clinical features and outcomes of hyperglycemic hyperosmolar syndrome: a retrospective study at a Japanese university hospital

**DOI:** 10.1007/s13340-025-00823-z

**Published:** 2025-06-16

**Authors:** Taku Fujiya, Kotoko Iwafuchi, Takuho Itasaka, Keita Ujiie, Taichi Watanabe, Yuichiro Munakata, Yasuhiro Tanji, Shojiro Sawada

**Affiliations:** Division of Metabolism and Diabetes, Faculty of Medicine, Tohoku Medical and Pharmaceutical University, Fukumuro 1-12-1, Miyagino-ku, Sendai, 983-8512 Japan

**Keywords:** Hyperglycemic hyperosmolar syndrome, Type 2 diabetes, Metabolic acidosis, 30-day mortality

## Abstract

**Background:**

Hyperglycemic hyperosmolar syndrome (HHS) is a severe complication of diabetes, and is triggered by infection, use of corticosteroids, non-adherence to medications, and acute diseases. Despite advances in diabetes management, the clinical features and outcomes of patients with HHS remain unclear. This study aimed to investigate the clinical characteristics and prognoses of patients with HHS at a university hospital in Japan.

**Methods:**

This retrospective cohort study analyzed 84 consecutive patients diagnosed with HHS between 2018 and 2023. Patients with HHS were classified into “isolated HHS” and “mixed diabetic ketoacidosis (DKA)/HHS” groups based on established criteria. A subgroup analysis further divided the isolated HHS group into those with and without metabolic acidosis. Clinical data were collected from electronic medical records.

**Results:**

The median age of patients with HHS was 75 years, with infections (51.2%) being the most common trigger. Approximately 70% of patients did not receive diabetes medication. The isolated HHS group had a significantly higher 30-day mortality rate (26.0%) than the mixed DKA/HHS group (0%). In the isolated HHS group, those with metabolic acidosis showed markedly worse outcomes, with a 30-day mortality rate of 54.6% versus 20.7% in those without acidosis.

**Conclusion:**

HHS predominantly affects elderly patients and is often associated with poor diabetes management. The isolated HHS group had a worse prognosis than the mixed DKA/HHS group, and the presence of metabolic acidosis other than ketoacidosis significantly increased mortality. Regular blood glucose monitoring and appropriate diabetes medications are crucial for preventing the development of HHS.

## Introduction

Hyperglycemic emergencies are serious acute complications of diabetes, which include diabetic ketoacidosis (DKA) and hyperglycemic hyperosmolar syndrome (HHS) [1]. Common causes of hyperglycemic emergencies include infection, nonadherence to medications, and acute diseases such as stroke, myocardial infarction, and trauma. Additionally, some medications such as corticosteroids and antipsychotics have been reported to impair glucose tolerance [2]. Patients with HHS are generally older than those with DKA; however, HHS may increasingly be seen in young adults as the first presentation of newly diagnosed diabetes [3]. HHS is more likely to occur in type 2 diabetes than in type 1 diabetes [4], and often develops over many days; consequently, dehydration and metabolic disturbances are usually more extreme than those with DKA [5]. The mortality rate of HHS is high (10–20%), and is estimated to be 10 times higher than that of DKA [1, 6]. Furthermore, the mortality rate of elderly or unconscious patients with HHS could be even higher [7, 8]. Consequently, HHS is one of the most deleterious complications of diabetes even in the present day [6]. Clinical overlap between HHS and DKA has been reported in more than one-third of patients with hyperglycemic crises [9, 10]. However, the exact features and prognosis of HHS remain unclear. Therefore, in this study, we aimed to investigate the clinical characteristics of patients with HHS at a university hospital in Japan and identify the factors associated with HHS prognosis and outcomes.

## Materials and methods

### Study population and design

**Fig. 1:**
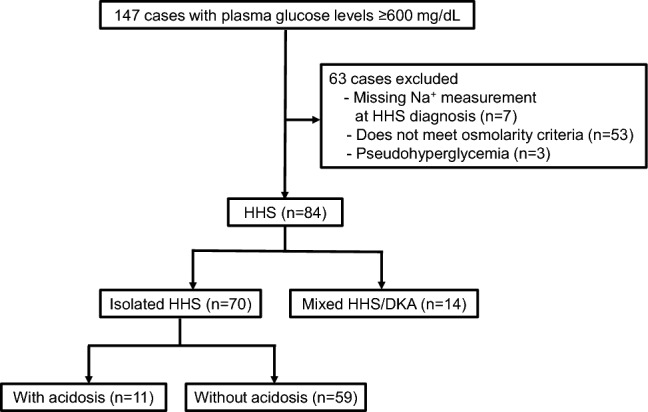
Flowchart of participant selection

This retrospective cohort study was conducted to investigate the clinical characteristics of patients with HHS at Tohoku Medical and Pharmaceutical University Hospital between January 2018 and December 2023. All patients with HHS were identified using their electronic medical records. We included patients with plasma glucose level ≥ 600 mg/dL and hyperosmolarity. The definition of hyperosmolarity in the present study was based on the diagnostic criteria for HHS published by the American Diabetes Association (ADA) in 2024, with effective serum osmolality > 300 mOsm/kg or total serum osmolality > 320 mOsm/kg [11]. Next, as shown in Fig. 1, we divided HHS into isolated HHS and mixed DKA/HHS, defined. Isolated HHS was defined as hyperglycemia (plasma glucose level ≥ 600 mg/dL), hyperosmolarity (effective serum osmolality > 300 mOsm/kg or total serum osmolality > 320 mOsm/kg), and absence of significant ketonemia (β-hydroxybutyrate < 3.0 mmol/L or urine ketone strip < 2 +). The effective serum osmolality was calculated as: (2 × Na^+^ [mmol/L] + glucose [mg/dL]/18). Total serum osmolality was calculated as: (2 × Na^+^ [mmol/L] + glucose [mg/dL]/18 + urea [mg/dL] / 2.8). Mixed DKA/HHS was defined as ketosis (β-hydroxybutyrate ≥ 3.0 mmol/L or urine ketone strip ≥ 2 +), metabolic acidosis (pH < 7.3 and/or HCO_3_^−^ < 18 mmol/L), hyperglycemia (plasma glucose level ≥ 600 mg/dL), and hyperosmolarity (effective serum osmolality > 300 mOsm/kg or total serum osmolality > 320 mOsm/kg). Lactic acidosis was defined as blood lactate level > 5 mmol/L, pH < 7.35, and HCO_3_^−^ < 20 mmol/L [12].

### Data collection

The following data were extracted from electronic medical records: age, sex, height, weight, body mass index (BMI), history of diabetes, type of diabetes diagnosed, coexisting diseases, living environment, diabetes medications before the onset of hyperglycemic crises, precipitating causes of hyperglycemic crises, consciousness level (Japan Coma Scale [JCS]), and length of hospital stay. The following biochemical parameters were collected at the time of HHS diagnosis: levels of plasma glucose, glycated hemoglobin (HbA1c), pH, HCO_3_^−^, sodium (Na^+^), potassium (K^+^), chloride (Cl^−^), lactate, creatinine (Cr), urea nitrogen (UN), β-hydroxybutyrate, and ketone bodies in the urine test strip. The estimated glomerular filtration rate (eGFR) was calculated using the formula recommended by the Japanese Society of Nephrology [13], derived from the Modification of Diet in Renal Disease Study Group.

### Subgroup

**Fig. 2:**
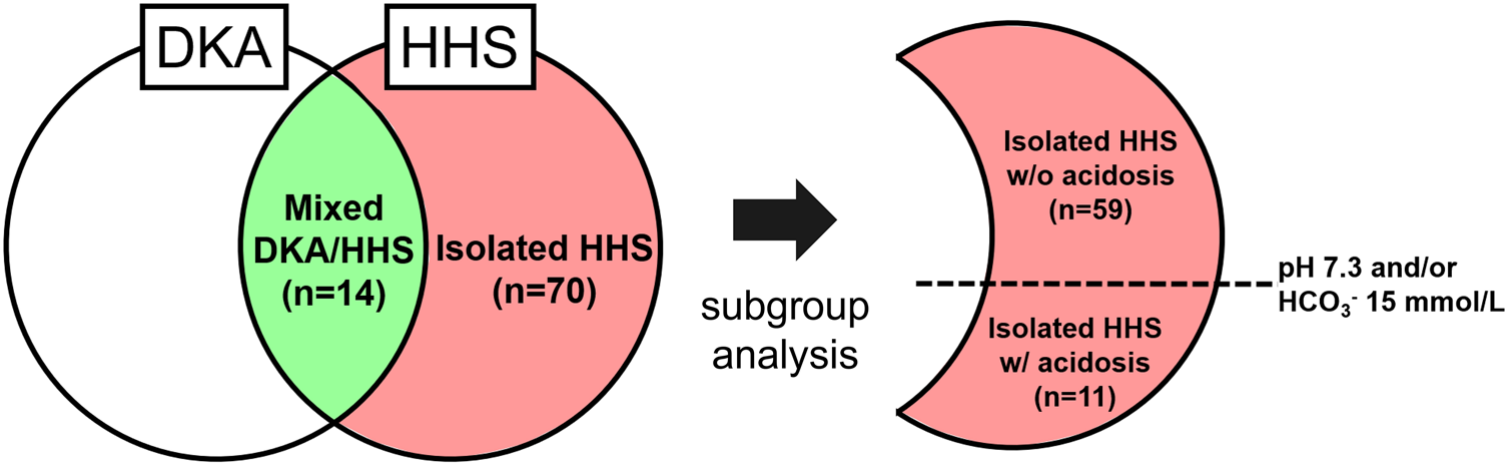
Relationship diagram of mixed DKA/HHS, isolated HHS without acidosis, and isolated HHS with acidosis. HHS, hyperglycemic hyperosmolar syndrome; DKA, diabetic ketoacidosis; w/o, without; w/, with

The diagnostic criteria for HHS in the American Diabetes Association (ADA) [11] include the absence of significant acidosis (pH ≥ 7.3 and HCO_3_^−^ ≥ 15 mmol/L). However, patients with HHS sometimes develop concomitant metabolic acidosis other than ketoacidosis due to exacerbation of the underlying acute illness or acute kidney injury. Then, as shown in Fig. 2, patients with isolated HHS were further divided into two subgroups: those without significant acidosis (pH ≥ 7.3 and HCO_3_^−^ ≥ 15 mmol/L) and those with significant acidosis (pH < 7.3 and/or HCO_3_^−^ < 15 mmol/L). In the present study, the former referred to as “isolated HHS w/o acidosis” and the latter as “isolated HHS w/ acidosis”.

### Statistical Methods

Data management and statistical analyses were performed using the JMP 14.2.0 software (SAS Institute Inc., Cary, NC 27513, USA). Categorical variables are presented as counts (percentages). Continuous variables followed a non-normal distribution and are presented as medians (first quartiles [Q1]; third quartiles [Q3]). Inter-group comparisons were conducted using Fisher's exact test for categorical variables and the Mann–Whitney U test for continuous variables. The 30-day survival curves for the two groups were estimated using the Kaplan–Meier method and compared using the log-rank test. Patients lost to follow-up before 30 days were censored on the last date of contact. The Kaplan–Meier survival curves in the subgroup analysis showed divergence over time between groups, indicating a potential violation of the proportional hazard assumption. Therefore, Cox proportional hazards analysis was not performed. Instead, logistic regression analysis adjusted for age and sex was conducted to estimate the odds ratio and 95% confidence intervals (CI) for death within 30 days. Statistical significance was set at a *P*-value of less than 0.05.

**Fig. 3:**
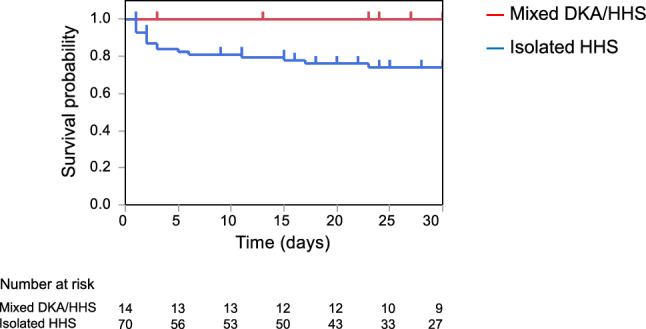
The 30-day mortality rate in the isolated HHS and mixed HHS/DKA groups. Kaplan–Meier estimates of survival probability for isolated HHS and mixed HHS/DKA (log-rank test, *p* = 0.0452). HHS, hyperglycemic hyperosmolar syndrome; DKA, diabetic ketoacidosis

## Results

### Patient characteristics

The clinical characteristics of the patients are summarized in Table 1. This study included 84 patients with diabetes who were admitted to Tohoku Medical and Pharmaceutical University Hospital due to hyperglycemic crisis and met the diagnostic criteria for HHS. The median patient age was 75 years, and 53.6% of the patients were male. Patients with known type 1 diabetes accounted for 13.1% of the cohort, known type 2 diabetes accounted for 64.3%, and newly diagnosed diabetes at admission accounted for 17.9%. Unexpectedly, 69.1% of the patients were not taking diabetes medications at the time of admission, i.e., many patients had been diagnosed with diabetes in the past but were not currently being treated or had self-neglected medication. Regarding the cause of HHS development, infection was the most common cause at 51.2%, followed by corticosteroid use at 21.4%, enteral nutrition at 14.3%, oral antipsychotic use at 10.7%, total parenteral nutrition at 6.0%, stroke at 6.0%, ischemic heart disease at 3.6%, heart failure at 2.4%, aortic dissection at 1.2%, pulmonary embolism at 1.2%, and non-occlusive mesenteric ischemia (NOMI) at 1.2%. Among infectious diseases, pneumonia accounted for 53.5% and urinary tract infections for 20.9% of cases. Other infections included two cases of infected decubitus ulcers and one case each of iliopsoas muscle abscess, lower extremity cellulitis, cholecystitis, and lower extremity gangrene. Prior to admission to our hospital, 67.9% lived with their family, 13.1% lived alone, 17.9% lived in a nursing home, and 1.2% lived in another hospital. The level of consciousness at the time of HHS diagnosis was assessed using the JCS. The JCS is widely used in Japanese clinical practice, although the Glasgow Coma Scale (GCS) is the international gold standard for assessing the level of consciousness. The JCS is based on the degree of arousal and is categorized into three stages: grade 1, aroused; grade 2, aroused in response to stimuli; and grade 3, not aroused even when stimulated. Grade 0 was defined as clear consciousness. The JCS and GCS have been reported to be strongly correlated each other [14]. Among all HHS cases, 25.0%, 30.0%, 16.7%, and 28.6% had JCS grades 0, 1, 2, and 3, respectively. The mean length of hospital stay from HHS diagnosis to discharge was 23 days.

**Table 1:** Clinical characteristics of patients with HHS

	All HHS (*n* = 84)		Isolated HHS (*n* = 70)		Mixed DKA/HHS (*n* = 14)		*p*-value	
Age, years	75 (64–82)		75 (66–82)		80 (57–86)		0.8712	
Male, n (%)	45 (53.6)		40 (57.1)		5 (35.7)		0.1572	
Body mass index, kg/ m^2^	21.3 (18.1–23.3)		21.1 (18.5–22.8)		22.4 (17.8–24.8)		0.3242	
Diabetes status at admission, n (%)								
Known type 1 diabetes	11 (13.1)		5 (7.1)		6 (42.9)		0.0021	
Known type 2 diabetes	54 (64.3)		50 (71.4)		4 (28.6)		0.0045	
Known pancreatogenic diabetes	1 (1.2)		1 (1.4)		0 (0.0)		1.0000	
Known other diabetes	3 (3.6)		3 (4.3)		0 (0.0)		1.0000	
Newly diagnosed diabetes	15 (17.9)		11 (15.7)		4 (28.6)		0.2642	
Diabetes medication at admission, n (%)								
No medication	58 (69.1)		48 (68.6)		10 (71.4)		1.0000	
Insulin	14 (16.7)		10 (14.3)		4 (28.6)		0.2374	
GLP-1 receptor agonist	3 (4.6)		3 (4.3)		0 (0)		1.0000	
DPP-4 inhibitor	15 (17.9)		13 (18.6)		2 (14.3)		1.0000	
SGLT2 inhibitor	4 (4.8)		4 (5.7)		0 (0)		1.0000	
Sulfonylurea	7 (8.3)		7 (10.0)		0 (0)		0.5947	
Metformin	14 (16.7)		13 (18.6)		1 (7.1)		0.4468	
α-Glucosidase inhibitor	6 (7.1)		6 (8.6)		0 (0)		0.5831	
Glinide	1 (1.2)		1 (1.4)		0 (0)		1.0000	
Comorbidities, n (%)								
Cardio-cerebrovascular disease	35 (41.7)		30 (42.9)		5 (35.7)		0.7694	
Cancer	29 (34.5)		25 (35.7)		4 (28.6)		0.7621	
Dementia	19 (22.6)		17 (24.3)		2 (14.3)		0.5081	
Precipitating cause, n (%)								
Infectious disease		43 (51.2)		36 (51.4)		7 (50.0)		1.0000
Steroid use		18 (21.4)		16 (22.9)		2 (14.3)		0.7238
Antipsychotic drug use		9 (10.7)		7 (10.0)		2 (14.3)		0.6410
Enteral nutrition		12 (14.3)		12 (17.1)		0 (0.0)		0.2029
Total parental nutrition		5 (6.0)		5 (7.1)		0 (0.0)		0.5842
Ischemic heart disease		3 (3.6)		3 (4.3)		0 (0.0)		1.000
Heart failure		2 (2.4)		1 (1.4)		1 (7.1)		0.3072
Aortic dissection		1 (1.2)		1 (1.4)		0 (0.0)		1.000
Pulmonary embolism		1 (1.2)		1 (1.4)		0 (0.0)		1.000
Stroke		5 (6.0)		4 (5.7)		1 (7.1)		1.000
Non-occlusive mesenteric ischemia		1 (1.2)		1 (1.4)		0 (0.0)		1.000
Living environment prior to admission, n (%)								
Living alone		11 (13.1)		11 (15.7)		0 (0.0)		0.1974
Living with family		57 (67.9)		43 (62.3)		14 (100)		0.0038
Nursing home		15 (17.9)		15 (21.4)		0 (0.0)		0.0643
Other hospital		1 (1.2)		1 (1.4)		0 (0.0)		1.0000
JCS grade at admission, n (%)								
Grade 0, n (%)		21 (25.0)		20 (28.6)		1 (7.1)		0.0128
Grade 1, n (%)		25 (30.0)		18 (25.7)		7 (50.0)		
Grade 2, n (%)		14 (16.7)		9 (12.9)		5 (35.7)		
Grade 3, n (%)		24 (28.6)		23 (32.9)		1 (7.1)		
Length of hospital stay, days		23 (11–37)		21 (5–34)		36 (24–46)		0.0261

Data are presented as medians (inter-quantile range (IQR)) for continuous variables and % for categorical variables

HHS, hyperglycemic hyperosmolar syndrome; DKA, diabetic ketoacidosis; GLP, glucagon-like peptide-1; DPP, dipeptidyl peptidase; SGLT, sodium glucose co-transporter; JCS, Japan Coma Scale

Of the 84 patients with HHS, 70 were in the isolated HHS group and 14 were in the mixed DKA/HHS group. The diabetes status at admission in the isolated HHS group was predominantly known type 2 diabetes (71.4%). In contrast, in the mixed DKA/HHS group, the proportion of known type 1 diabetes was relatively high (42.9%). As for the living environment prior to hospitalization, 62.3% of the patients in the isolated HHS group were living with family, whereas all the patients in the mixed DKA/HHS group were living with family. The level of consciousness was JCS grade 3 in 32.9% of the patients in the isolated HHS group and 7.1% in the mixed DKA/HHS group. Thus, the isolated HHS group had a higher percentage of patients with severe levels of consciousness on admission than the mixed DKA/HHS group.

**Fig. 4:**
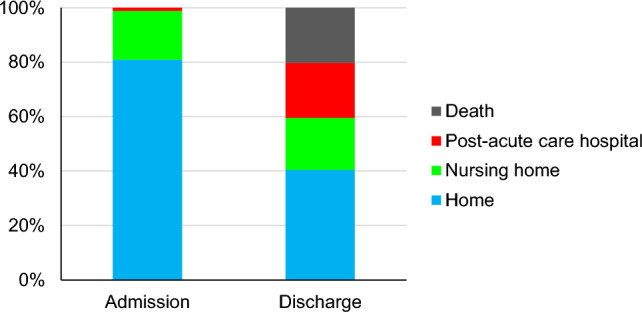
Living environments. Living environments (post-acute care hospital, nursing home, home) before and after hospitalization

### Laboratory data

In all patients with HHS, the glucose level was 765 mg/dL, the HbA1c level was 9.8%, and the effective osmolality was 320 mOsm/kg. The UN level was 47 mg/dL, creatinine level was 1.4 mg/dL, and the UN/Cr ratio was 33.6, indicating a dehydrated state. The eGFR was 33.5 ml/min /1.73 m^2^, indicating poor renal function. The lactate levels were as high as 2.8 mmol/L. In the isolated HHS group, the plasma Cl^−^, pH, and HCO_3_^−^ levels were significantly higher than in the mixed DKA/HHS group, while the K^+^ levels were significantly lower in the isolated HHS group than in the mixed DKA/HHS group (Table 2).

**Table 2:** Laboratory data of patients with HHS

	All HHS (*n* = 84)	Isolated HHS (*n* = 70)	Mixed DKA/HHS (*n* = 14)	*p*-value
Glucose, mg/dL	765 (675–936)	760 (675–906)	823 (653–1020)	0.6269
HbA1c, %	9.8 (8.4–12.9)	9.7 (8.5–12.8)	10.9 (8.1–14.4)	0.4748
Na, mmol/L	136 (130–149)	137 (131–151)	133 (127–142)	0.0679
K, mmol/L	4.8 (4.4–5.5)	4.8 (4.3–5.1)	5.8 (5.1–6.3)	0.0004
Cl, mmol/L	98 (91–109)	100 (92–111)	95 (86–102)	0.0275
Effective osmolarity, mOsm/kg	320 (304–342)	321 (304–353)	313 (304–325)	0.2367
Urea nitrogen (mg/dL)	47 (29–73)	47 (26–72)	50.0 (33–86)	0.4423
Creatinine (mg/dL)	1.4 (0.9–2.2)	1.4 (0.9–2.0)	1.6 (1.0–3.0)	0.5169
eGFR (ml/min/1.73 m^2^)	33.5 (22.1–55.5)	32.4 (22.6–63.3)	34.3 (15.7–60.0)	0.4282
pH	7.35 (7.22–7.41)	7.38 (7.31–7.43)	7.18 (7.06–7.24)	< 0.0001
HCO_3_^−^, mmol/L	21.0 (12.7–27.8)	23.4 (16.7–28.6)	7.6 (3.3–10.6)	< 0.0001
Lactate, mmol/L	2.8 (1.8–4.2)	2.8 (1.9–4.3)	2.3 (1.8–2.8)	0.1272

Data are presented as medians (inter-quantile range (IQR)) for continuous variables

HHS, hyperglycemic hyperosmolar syndreome; DKA, diabetic ketoacidosis; eGFR, estimated glomerular filtration rate

### Mortality

All 17 observed deaths occurred in the isolated HHS group while no deaths were occurred in the mixed DKA/HHS group. The causes of death were as follows: seven cases of pneumonia, three cases of ischemic heart disease, three of sepsis, and one each of pulmonary embolism, NOMI, cerebral infarction, and malignant disease (sigmoid colon cancer, liver metastasis, and lung metastasis). The 30-day cumulative mortality in all patients with HHS patients was 21.5%. As shown in Fig. 3, the 30-day mortality rate in the isolated HHS group was 26.0%, whereas that in the mixed DKA/HHS group was 0%. The Kaplan–Meier plot showed a significantly increased 30-day mortality in the isolated HHS group compared with that in the mixed DKA/HHS group (log-rank test, *p* = 0.0452). No deaths occurred in the mixed DKA/HHS group during the follow-up period; therefore, Cox proportional hazard analysis was not performed. Thus, the isolated HHS group had a worse prognosis than the mixed DKA/HHS group.

### Living environments

We investigated the discharge destinations of the patients with HHS who survived (Fig. 4). Of the 84 patients included in the study, 68 were admitted from home, 15 from a nursing home, and 1 from another hospital. During hospitalization, 17 patients died, 17 were transferred to a post-acute care hospital, 16 were discharged to a nursing home, and 34 were discharged to home. Notably, among patients with HHS who were admitted from home, approximately half of the patients were unable to return to their home following discharge, highlighting the substantial impact and severity of HHS.

**Fig. 5:**
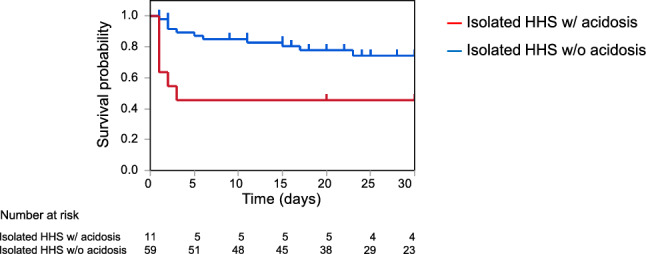
The 30-day mortality in the isolated HHS without acidosis and the isolated HHS with acidosis groups. Kaplan–Meier estimates of survival probability for patients with isolated HHS without acidosis and isolated HHS with acidosis groups (log-rank test, *p* = 0.0025). HHS, hyperglycemic hyperosmolar syndrome

### Subgroup

Of the 70 patients with isolated HHS, 11 had acidosis (Tables 3 and 4). The patients in the group with acidosis were younger than those in the group without acidosis. In the group with acidosis, 45.5% of the patients were newly diagnosed with diabetes, which was significantly higher than the 10.2% in the group without acidosis. The group with acidosis had fewer infectious diseases as a precipitating cause than the group without acidosis. However, there were more serious acute illness; three cases of ischemic heart disease and one each pulmonary embolism, cerebral infarction, NOMI in the group with acidosis. The group with acidosis had a higher percentage of JCS grade 3 than the group without acidosis. Prior to hospitalization, 52.4% of the patients in the group without acidosis were living with family, whereas 100% of patients in the group with acidosis were living with family. The K^+^ and lactate levels in the group with acidosis were higher than those in the group without acidosis. In contrast, the HCO_3_^−^ levels in the acidosis group were lower than those in the group without acidosis.

**Table 3:** Clinical characteristics of patients with isolated HHS divided by the presence or absence of acidosis

	Isolated HHS (*n* = 70)		Isolated HHS with acidosis (*n* = 11)		Isolated HHS without acidosis (*n* = 59)		*p*-value
Age, years	75 (66–82)		60 (49–72)		76 (68–84)		0.0056
Male, n (%)	40 (57.1)		6 (54.6)		34 (57.6)		1.0000
Body mass index, kg/ m^2^	21.1 (18.5–22.8)		21.9 (18.7–29.2)		20.5 (17.7–22.7)		0.3141
Diabetes status at admission, n (%)							
Known type 1 diabetes	5 (7.1)		2 (18.2)		3 (5.1)		0.1727
Known type 2 diabetes	50 (71.4)		4 (36.4)		46 (78.0)		0.0095
Known pancreatogenic diabetes	1 (1.4)		0 (0.0)		1 (1.7)		1.0000
Known other diabetes	3 (4.3)		0 (0.0)		3 (5.1)		1.0000
Newly diagnosed diabetes	11 (15.7)		5 (45.5)		6 (10.2)		0.0108
Diabetes medication at admission, n (%)							
No medication	48 (68.6)		8 (72.7)		40 (67.8)		1.0000
Insulin	10 (14.3)		2 (18.2)		8 (13.6)		0.6516
GLP-1 receptor agonist	3 (4.3)		0 (0)		3 (5.1)		1.0000
DPP-4 inhibitor	13 (18.6)		1 (9.1)		12 (20.3)		0.6757
SGLT2 inhibitor	4 (5.7)		0 (0)		4 (6.8)		1.0000
Sulfonylurea	7 (10.0)		1 (9.1)		6 (10.2)		1.0000
Metformin	13 (18.6)		3 (27.3)		10 (17.0)		0.4163
α-Glucosidase inhibitor	6 (8.6)		2 (18.2)		4 (6.8)		0.2363
Glinide	1 (1.4)		0 (0)		1 (1.7)		1.0000
Comorbidities, n (%)							
Cardio-cerebrovascular disease	30 (42.9)		3 (27.3)		27 (45.8)		0.3305
Cancer	25 (35.7)		2 (18.2)		23 (39.0)		0.3056
Dementia	17 (24.3)		1 (9.1)		16 (27.1)		0.2734
Precipitating cause, n (%)							
Infectious disease		36 (51.4)		2 (18.2)		34 (57.6)	0.0219
Steroid use		16 (22.9)		1 (9.1)		15 (25.4)	0.4358
Antipsychotic drug use		7 (10.0)		0 (0.0)		7 (11.9)	0.5866
Enteral nutrition		12 (17.1)		2 (18.2)		10 (17.0)	1.0000
Total parental nutrition		5 (7.1)		1 (9.1)		4 (6.8)	1.0000
Ischemic heart disease		3 (4.3)		3 (27.3)		0 (0.0)	0.0030
Heart failure		1 (1.4)		0 (0.0)		1 (1.7)	1.0000
Aortic dissection		1 (1.4)		0 (0.0)		1 (1.7)	1.0000
Pulmonary embolism		1 (1.4)		1 (9.1)		0 (0.0)	0.1571
Stroke		4 (5.7)		1 (9.1)		3 (5.1)	0.5036
Non-occlusive mesenteric ischemia		1 (1.4)		1 (9.1)		0 (0.0)	0.1571
Living environment prior to admission, n (%)							
Living alone		11 (15.7)		0 (0.0)		11 (18.6)	0.1921
Living with family		43 (61.4)		11 (100)		32 (52.4)	0.0048
Nursing home		15 (21.4)		0 (0.0)		15 (25.4)	0.1047
Other hospital		1 (1.4)		0 (0.0)		1 (1.7)	1.0000
JCS grade at admission, n (%)							
Grade 0, n (%)		20 (28.6)		0 (0.0)		20 (33.9)	0.0149
Grade 1, n (%)		18 (25.7)		2 (18.2)		16 (27.1)	
Grade 2, n (%)		9 (12.9)		1 (9.1)		8 (13.6)	
Grade 3, n (%)		23 (32.9)		8 (72.7)		15 (25.4)	
Length of hospital stay, days		21 (5–34)		3 (1–36)		22 (11–31)	0.2355

Data are presented as medians (inter-quantile range (IQR)) for continuous variables and % for categorical variables

HHS, hyperglycemic hyperosmolar syndrome; DKA, diabetic ketoacidosis; GLP, glucagon-like peptide-1; DPP, dipeptidyl peptidase; SGLT, sodium glucose co-transporter; JCS, Japan Coma Scale

**Table 4:** Laboratory data of patients with isolated HHS divided by the presence or absence of acidosis

	Isolated HHS (*n* = 70)	Isolated HHS with acidosis (*n* = 11)	Isolated HHS without acidosis (*n* = 59)	*p*-value
Glucose, mg/dL	760 (675–906)	964 (627–1216)	739 (675–867)	0.1014
HbA1c, %	9.7 (8.5–12.8)	10.2 (7.5–13.0)	9.6 (8.5–12.7)	0.8512
Na, mmol/L	137 (131–151)	136 (131–148)	137 (131–153)	0.5775
K, mmol/L	4.8 (4.3–5.1)	5.7 (5.2–8.2)	4.7 (4.3–5.0)	0.0009
Cl, mmol/L	100 (92–111)	97 (90–110)	100 (93–111)	0.3615
Effective osmolarity, mOsm/kg	321 (304–353)	331 (308–354)	320 (302–352)	0.5079
Urea nitrogen (mg/dL)	47 (26–72)	56 (18–90)	46 (29–69)	0.9421
Creatinine (mg/dL)	1.4 (0.9–2.0)	2.0 (1.0–4.1)	1.4 (0.8–2.0)	0.0786
eGFR (ml/min/1.73 m^2^)	32.4 (22.8–63.3)	21.0 (14.0–43.0)	34.0 (24.0–65.9)	0.0813
pH	7.35 (7.22–7.41)	7.06 (6.70–7.21)	7.39 (7.35–7.44)	< 0.0001
HCO_3_^−^, mmol/L	23.4 (16.7–28.6)	12.6 (6.3–16.1)	26.0 (21.3–29.5)	< 0.0001
Lactate, mmol/L	2.8 (1.9–4.3)	14.6 (9.9–23.0)	2.4 (1.8–3.2)	< 0.0001

Data are presented as medians (inter-quantile range (IQR)) for continuous variables

HHS, hyperglycemic hyperosmolar syndrome; DKA, diabetic ketoacidosis; eGFR, estimated glomerular filtration rate

In the isolated HHS group, six deaths were occurred in those with acidosis and 11 in those without acidosis. As shown in Fig. 5, the isolated HHS with acidosis group had a higher 30-day mortality rate (54.6%) than the group without acidosis (20.7%). The Kaplan–Meier survival curves showed significantly higher 30-day mortality in the isolated HHS group with acidosis than in the isolated HHS group without acidosis (log-rank test, *p* = 0.0025). In the isolated HHS with acidosis group, all deaths occurred within the first 3 days of admission, with no additional deaths observed thereafter. Conversely, in the HHS without acidosis group, mortality events continued to occur throughout the 30-day observation period. Thus, the Kaplan–Meier survival curves showed divergence over time between the groups, indicating a potential violation of the proportional hazard assumption. Therefore, we conducted a logistic regression analysis with death within 30 days as the dependent variable. In an age- and sex-adjusted logistic regression analysis, the presence of acidosis was significantly associated with mortality, with an odds ratio of 8.08 (95% CI, 1.63–40.2; *p* = 0.011). The causes of death in the isolated HHS group with acidosis were as follows; three cases of ischemic heart disease and one each of pulmonary embolism, NOMI, cerebral infarction. All six of these patients were accompanied by lactic acidosis. Thus, the isolated HHS group with acidosis had a worse prognosis than the isolated HHS group without acidosis.

## Discussion

The major findings of this study included the following. First, patients with HHS were older (median age: 75 years), and infection was the most common trigger, followed by steroid use, enteral nutrition, oral antipsychotic use, and total parenteral nutrition. In addition, approximately two-thirds of the patients were not taking diabetes medication at the onset of HHS. Second, of the 84 patients with HHS, 70 were classified into the isolated HHS group and 14 into the mixed DKA/HHS group. The 30-day mortality rate was significantly higher in the isolated HHS group than in the mixed DKA/HHS group. Third, among the isolated HHS group, those with metabolic acidosis other than ketoacidosis had a particularly poor prognosis.

In our study, 17.9% of patients were newly diagnosed with diabetes at the onset of HHS. The remaining 82.1% had known type 1, type 2, pancreatogenic or other diabetes. Kitabchi et al. reported that the proportion of patients with undiagnosed diabetes before HHS was 20% [15], which is consistent with the results of our study. Of all 84 patients with HHS, 69 (82.1%) were previously diagnosed with diabetes, but only 26 of the 69 patients were receiving diabetes medications, while the remaining 43 were not. Therefore, regular blood glucose monitoring and appropriate diabetes medications in outpatient clinics may be important to prevent the development of HHS. Infections have been widely reported as the primary precipitating factors of HHS, with pneumonia being the most common, followed by urinary tract infections [16]. Consistent with previous reports, our study identified infection as the leading cause of HHS, accounting for 51.2% of all HHS cases. Therefore, when managing patients with HHS, it may be essential to consider antibiotic therapy in parallel with insulin-based blood glucose control, considering the possibility of underlying infections.

In our study, the 30-day mortality rate for HHS was 21.5%, which is higher than the 10–20% reported in previous studies [17–20]. In a hospital-based retrospective cohort study in Taiwan [17], involving 978 patients with HHS hospitalized from 1991 to 2005, the 28-day mortality rate was 18.83%. The mean age of these patients was 67.9 years, 7 years younger than our sample. When stratified by age, the mortality rate was reported to be only 3.6% for those aged below 45 years, 5.8% for those aged 45–69 years, and 17.9% for those over 69 years. Thus, age had a strong influence on the HHS mortality rate. Another study, the Danish nationwide residence-based cohort study [18], comprising 634 adult patients with HHS from 2016 to 2018, reported a hospitalization mortality rate of 14.0%, the mean age of the patients was 69 years, 6 years younger than our patients. In addition, the comorbidity rate of dementia in that study was 5.5%, lower than the 22.6% observed in our study, while the comorbidity rate of cancer was 12.9%, lower than 34.5% observed in our study. Therefore, the higher mortality rate in our study may have been due to older age and higher rates of comorbidities. In a recent multicenter retrospective cohort study of 21 acute care hospitals in Japan on hyperglycemic crises [21], 226 adult hospitalized patients with HHS from 2012 to 2016 were analyzed. The reported in-hospital mortality was 7.1%. The mean age of the patients was 71.9 years, 3 years younger than our sample. Infectious diseases were identified as a precipitating factor for HHS in 42.7% of the patients, lower than the 51.2% observed in our study. The steroid use rate was 6.2%, which is also lower than the 21.4% observed in our study. Furthermore, we compared the frequency of ischemic heart disease and stroke as precipitating factors for the development of HHS between our study and this Japanese multicenter retrospective cohort study. Notably, ischemic heart disease was identified as a precipitating factor for HHS in 1.3% of patients in that study, which is lower than the 3.6% observed in our cohort. The prevalence of stroke was 1.8%, which is also lower than the 6.0% observed in our cohort. Our hospital is a regional university hospital, where patients with serious emergencies are transported by the local emergency system. The differences in patient backgrounds may have contributed to the higher mortality rate in our study.

DKA is characterized by the triad of hyperglycemia, elevated blood and/or urine ketone body levels, and metabolic acidosis. The pathogenesis of DKA results from a combination of absolute or relative insulin deficiency and elevated levels of counteracting hormones (such as glucagon, epinephrine, norepinephrine, cortisol, and growth hormone). In contrast, HHS has residual insulin secretion to a degree that minimizes ketosis but fails to control hyperglycemia [9]. However, in clinical practice, one-third of patients with hyperglycemic crises show characteristics of both HHS and DKA [9, 10]. Therefore, in our study, HHS was separated into HHS without DKA and HHS with DKA, with the former being defined as “isolated HHS” and the latter as “mixed DKA/HHA”. Notably, the 30-day mortality rate in the isolated HHS group was higher than that in the mixed DKA/HHS group, indicating a significantly worse prognosis in the isolated HHS group. A report of the clinical profile of 144 patients admitted with hyperglycemic emergencies from 2012 to 2019 at a tertiary medical center in Kumamoto, Japan, showed that 87 were classified as having DKA alone, 38 as HHS alone, and 19 as mixed DKA/HHS. The in-hospital mortality rates were 5.7%, 13.2%, and 5.3%, respectively, with the HHS alone group having the poorest prognosis. The HHS alone group reported significantly higher serum Na^+^ levels and effective osmolarity than the mixed DKA/HHS group; hence, they may have been more dehydrated [20]. However, our study did not find differences in serum Na + levels and effective osmolarity between the isolated HHS and mixed DKA/HHS groups, although both tended to be higher in the isolated HHS than the mixed DKA/HHS group. The reason for the lack of statistically significant differences may be due to the small sample size. In the Danish national residence-based cohort study, 634 adult patients with HHS were divided into the isolated HHS group (*n* = 394) and mixed DKA/HHS group (*n* = 240) [18]. The in-hospital mortality rates were 17.3% and 8.8%, respectively, with a significantly worse prognosis in the isolated HHS group than in the mixed DKA/HHS group. Of note, the prevalence of type 1 diabetes in the Danish study was higher in the mixed DKA/HHS group (38.8%) than in the isolated HHS group (5.8%), a trend that was similarly observed in our study, with 42.9% in the mixed DKA/HHS group and 7.1% in the isolated HHS group. We hypothesized that the HHS of type 1 diabetes was more likely to be “mixed HHS + DKA” than “isolated HHS” and that the patients would be able to visit a hospital before their general condition became severe, since type 1 diabetes quickly progresses to DKA after insulin deficiency, whereas it takes several days for type 2 diabetes to progress to HHS. In fact, as shown in Table 1, the level of consciousness (JCS) at admission was significantly milder in the mixed DKA + HHS group, which had a higher proportion of type 1 diabetes, than in the isolated HHS group. Finally, although the difference was not statistically significant, steroid use was less frequent in the mixed DKA/HHS group than in the isolated HHS group. Additionally, the absence of enteral and total parental nutrition in the mixed DKA/HHS group may have contributed to the lower mortality rate observed in our study. As for the living environment prior to admission, the proportion of patients who lived with family was significantly higher in the mixed DKA/HHS group than in the isolated HHS group, but the reason for this is not clear. However, considering that 6 of the 14 patients with the mixed DKA/HHS had type 1 diabetes and were of older age, the need for continuous support in elderly patients with ketone-prone type 1 diabetes may have been a contributing factor to living with the family prior to admission.

Among the 84 patients with HHS in this study, only 34 individuals (40.5%) were discharged to their homes, indicating a substantial proportion of patients required alternative care arrangements post-discharge. In our study, approximately half (51.2%) of the patients with HHS had serious infections such as pneumonia and urinary tract infections that required long-term antibiotic therapy, resulting in an extended average hospital stay of 23 days. In elderly patients, the onset of HHS triggered a subsequent decline in activities of daily living and cognitive function, leading to increased disuse syndrome. These factors may have contributed to the difficulty of discharging patients to their homes in our study. Thus, even when death was averted, the functional prognosis was considered poor. Therefore, early recognition and prompt treatment are essential for improving outcomes in patients with HHS.

Finally, we found that the prognosis of HHS was particularly poor when it was accompanied by metabolic acidosis other than ketoacidosis. HHS is a serious condition that often coexists with acute illnesses, and metabolic acidosis can occur owing to disease progression or acute renal injury. In our study, 11 of 70 isolated HHS cases presented with metabolic acidosis other than ketoacidosis. Among the 11 patients, 6 died within 3 days of admission, and the causes of death were ischemic heart disease, pulmonary embolism, cerebral infarction, and NOMI, all of which were serious acute diseases. Hyperlactatemia reflects metabolic disturbances such as tissue hypoxia, anaerobic metabolism, and impaired lactate clearance. Hyperlactatemia has been reported to correlate with illness severity and is a predictor of mortality in trauma and non-trauma patients [22]. In our study, the lactate level in the isolated HHS with acidosis group was markedly higher at 14.6 mmol/L; hence, the higher mortality in this group was not surprising. Additionally, among the 11 patients with isolated HHS and metabolic acidosis, two were taking metformin. One patient died of ischemic heart disease on the first day of hospitalization. The other patient was in septic shock due to a urinary tract infection and was transferred to post-acute care hospital after 51 days. Then, the cause of their metabolic acidosis was thought to be a severe acute illness rather than metformin-associated lactic acidosis. Consequently, based on these results, the prognosis of patients with HHS with metabolic acidosis other than ketoacidosis is extremely poor. As for the living environment prior to admission, the proportion of patients who lived with family was significantly higher in the group with acidosis than in the group without acidosis, but the reason for this is not clear. Among the 11 patients in the isolated HHS with acidosis group, three experienced ischemic heart disease and one each experienced pulmonary embolism, cerebral infarction, and NOMI. All of these patients died within 3 days of hospitalization. In cases of severe and rapidly progressive acute arterial embolism, timely access to medical care would likely be impossible for patients living alone, and this may have contributed to the higher proportion of patients living with family observed in the group with acidosis.

This study had a few limitations. First, this was a single-center study with a small sample size. Second, this was a retrospective study with no long-term outcomes. Hence, future multicenter follow-up studies are needed to validate our findings in larger cohorts of patients.

In conclusion, our study demonstrated the characteristics and prognoses of patients with HHS at a single university hospital in Japan. HHS commonly occurred in elderly patients, many of whom were not receiving appropriate diabetes medications. Infection was the most common trigger for the development of HHS. Furthermore, isolated HHS had a worse prognosis than mixed DKA/HHS; moreover, the prognosis was worse when accompanied by metabolic acidosis other than ketoacidosis.

## Data Availability

The analyzed datasets are available from the corresponding author on reasonable request.
